# Seasonal variation in the occurrence of retinal vein occlusion: a 4-year cross-sectional study

**DOI:** 10.1186/s12886-020-01534-6

**Published:** 2020-07-06

**Authors:** Moe Matsuzawa, Yoshihito Sakanishi, Nobuyuki Ebihara

**Affiliations:** Department of Ophthalmology, Juntendo University Urayasu Hospital, 2-1-1 Tomioka, Urayasu, Chiba, Japan

**Keywords:** Retinal vein occlusion, Seasonality, Risk factors

## Abstract

**Background:**

Retinal vein occlusion (RVO) is a common retinal vascular disease that causes a loss of vision. Therefore, we investigated whether there is seasonal variation in the onset of RVO, to examine the possibility of preventing it.

**Methods:**

Patients with RVO who were treated at the Juntendo University Urayasu Hospital between April 2013 and March 2017 were included in this retrospective study. The season in which the RVO occurred was recorded for each case, and the cases were grouped into six 2-month periods based on the month of RVO, and classified by age, sex and hypertension status. The frequency of occurrence of RVO across seasons was compared using a chi-squared test.

**Results:**

A total of 348 patients with RVO presented during the study period, with information regarding the date of RVO onset. The cohort of 348 consisted of 167 males and 181 females who, overall, had a mean age of 64.0 years (range 17–96 years). The highest incidence of RVO onset was during January/February, with the lowest incidence during July/August. Patient age, sex and hypertension status did not influence the results.

**Conclusions:**

The seasonal onset of RVO tended to be higher in January/February and May/June, and lower in July/August. These findings suggest that eyecare professionals should be more vigilant in watching for the occurrence of RVO during winter and the rainy season, regardless of the patient’s sex, age or hypertension status.

## Background

Retinal vein occlusion (RVO) is a one of the vascular-occlusion diseases. The retinal vein narrows from mechanical pressure caused by arteriosclerosis, either within the arteriovenous crossing site in branch retinal vein occlusion (BRVO), or within the lamina cribrosa in central vein occlusion (CRVO), resulting in impairment of the venous blood flow. This can contribute to stasis, thrombosis and occlusion. RVO is the second most common retinal vascular disease after diabetic retinopathy, and causes a loss of vision due to macula edema [1–4].

Global epidemiological data indicate that the prevalence of RVO is 5.2/1000 [5] . Risk factors for RVO include hypertension, diabetes, increasing age, high body mass index, loss of protein C, loss of protein S, loss of antithrombin III and high antiphospholipid antibodies [6–8].

Cerebral infarction and myocardial infarction are vascular occlusions similar to RVO. While there are studies showing that the occurrence of cerebral infarction is highest in summer [9], there are also studies showing that it is highest in winter [10]. The risk of myocardial infarction has also been reported to be highest in winter [11]. These are arterial occlusion diseases, and some studies have shown that the risk of myocardial infarction and stroke was not high in BRVO patients [12, 13]. Conversely, there are studies showing that deep vein thrombosis (DVT), which is a venous occlusion, like RVO, is highest in winter [14, 15]. However, there has been limited investigation into whether climatic conditions are associated with the onset of RVO, and the existing findings show inconsistencies. In addition, the climate and seasonal changes are different between countries. Therefore, whether seasonal differences exist in different countries may provide a hint regarding the conditions underlying the seasonal differences.

In this study, we investigated whether seasonal climatic conditions had an impact on the onset of RVO in Japan. Japan has significant seasonal variation in climatic conditions such as temperature, atmospheric pressure, humidity, sunshine, rainfall and wind velocity. The impact of weather conditions on the onset of stroke or myocardial infarction has been examined, with temperature variation found to be a risk factor [11, 16]. The highest mortality rate is found in regions where the mean temperature is approximately 0 °C and the diurnal variance of the temperature is the biggest, ranging approximately 8–10 °C. In northeastern Japan, such climatic conditions in winter seem to be risk factors for stroke or myocardial infarction [16]. In contrast, there is limited evidence indicating whether climatic conditions represent risk factors for RVO onset. A Swedish study found a significant association between the onset of CRVO and the winter–spring period [17]. In London, the onset of CRVO showed significant cyclic variation, being most frequent in the months of September through February [18]. In Taiwan, the incidence of RVO is significantly associated with the seasons, with a peak in January [19]. All of these studies were conducted in the Northern Hemisphere, meaning that the seasonal variation in temperature would be similar to that experienced in Japan.

## Methods

### Subjects and classification

Diagnosis of RVO (either BRVO or CRVO) was made by confirming the presence of retinal hemorrhage and macular edema on fundus examination. Based on retrospective analysis of medical records, patients with RVO who were treated at the Juntendo University Urayasu Hospital between April 2013 and March 2017 were eligible for inclusion in this study. From those eligible patients, only patients with a defined date of disease onset were included in the final study cohort. The cases were grouped into six 2-month periods (January/February, March/April, May/June, July/August, September/October, November/December) based on the month of RVO onset and classified by age (< 65 years old or ≥ 65), sex and hypertension status. Hypertension status was established by patient self-report or based on medical records citing a systolic blood pressure of > 140 mmHg, for patients whose medical history was unknown.

### Statistical analysis

Data are presented as numbers or percentages. Whether the percentages of patients in each of the six groups were equal was assessed using a chi-squared test. The statistical analyses were conducted using SPSS version 26.0 (IBM, Chicago, IL, USA). A *P*-value < 0.05 was considered significant. Multiple comparisons were made by the bilateral exact binomial test using pwr package on R software version 3.3.3 and the *p*-values were adjusted using Benjamini & Hochberg procedure. Adjusted p-values are shown as q-values. An overall false discovery rate (FDR) under 0.05 was considered significant.

### Meteorological data

Average monthly temperatures of Japan and regions in the previous reports were obtained from the website of Japan Meteorological Agency (https://www.jma.go.jp/jma/indexe.html).

## Results

During the study period, 438 eyes were diagnosed with RVO. A total of 348 eyes with RVO, with known dates of onset, were examined. In cases where RVO was present in both eyes, the eye which presented earlier was included in the study results. Of the 348 patients with a known onset date, 281 patients were diagnosed with BRVO and 67 patients with CRVO. The mean age of the patients was 64.0 years (range 17–96 years).

### All cases

**Fig. 1:**
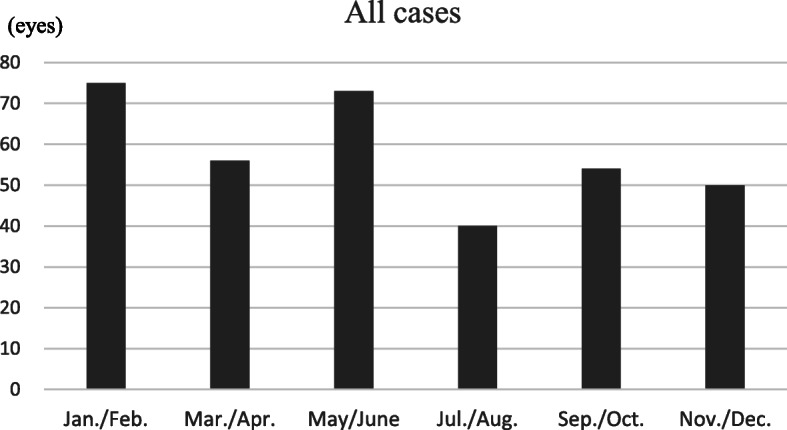
Onset periods for all 348 cases with a known month of retinal vein occlusion onset

Overall, there were 75 cases (21.6%) with an onset in the January/February period, 56 cases (16.1%) in March/April, 73 cases (21.0%) in May/June, 40 cases (11.5%) in July/August, 54 cases (15.5%) in September/October and 50 cases (14.4%) in November/December. The distribution of cases among the 6 groups was not even (*p* = 0.007), with a trough in July/August and peaks in January/February and May/June (Fig. 1). By multiple comparisons, two peaks were significantly higher than the trough (q = 0.019 each).

### Hypertension

**Fig. 2:**
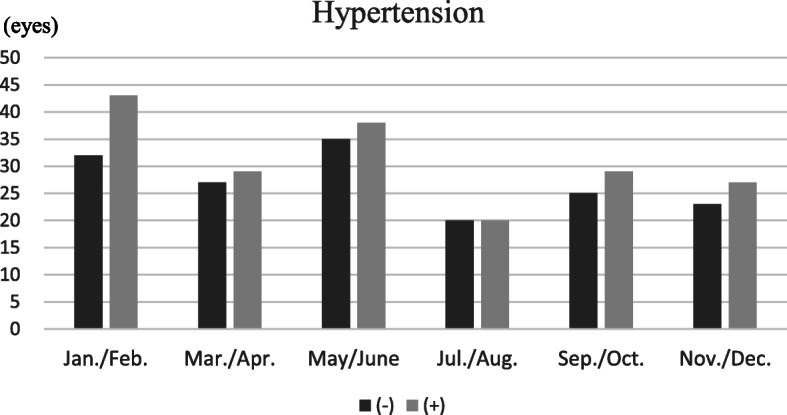
Retinal vein occlusion onset periods, based on the patients being classified as not having (dark bars) or having (grey bars) hypertension

There were 186 cases of RVO in patients with hypertension (53.4%) and 162 cases in patients without hypertension (46.6%). The highest incidence of RVO in patients with hypertension was found within the January/February period, with 43 cases (23%; see Fig. 2). The lowest incidence of RVO in patients with hypertension was found within the July/August period, with 120 cases (10.8%). The highest incidence of RVO in patients without hypertension was found to be in the May/June period, with 35 cases (21.6%; see Fig. 2). The lowest incidence of RVO in patients without hypertension was found within the July/August period, with 20 cases (12.3%). There were no statistically significant differences among the six periods with (*p* = 0.053) or without (*p* = 0.32) hypertension, but similar tendencies were observed for both groups.

### Sex

**Fig. 3:**
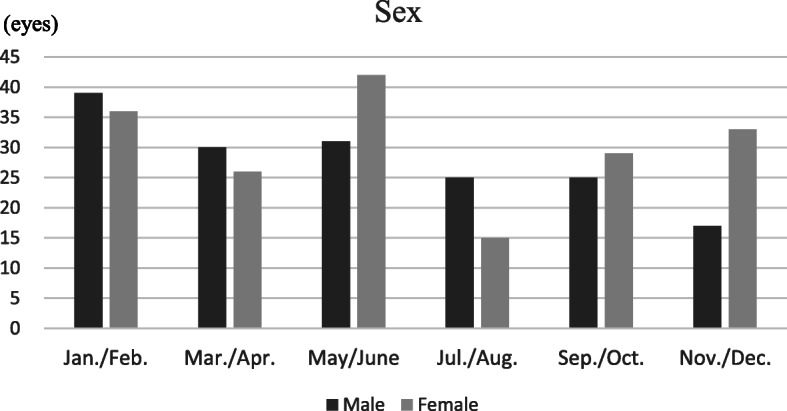
Retinal vein occlusion onset periods, with patients classified by sex (dark bars for males, grey bars for females)

Males accounted for 167 of the eyes with RVO (48.0%), with females accounting for the other 181 eyes (52.0%). Among the males, the highest incidence of RVO onset was found in the January/February period, with 39 eyes (23.3%; see Fig. 3), and the lowest incidence was found in the November/December period, with 17 eyes (10.2%). Among the females, the highest incidence of RVO onset was found in the May/June period, with 42 eyes (23.2%; see Fig. 3). The lowest incidence was found within the July/August period, with 15 eyes (8.3%). There were no statistically significant differences between the six periods, regardless of sex. Interestingly, females showed significant variation among six periods (*p* = 0.014) while males did not (*p* = 0.081). By multiple comparisons, two peaks were significantly higher than the trough in females (q = 0.035 and 0.0075 for January/February and May/June, respectively).

### Age

**Fig. 4:**
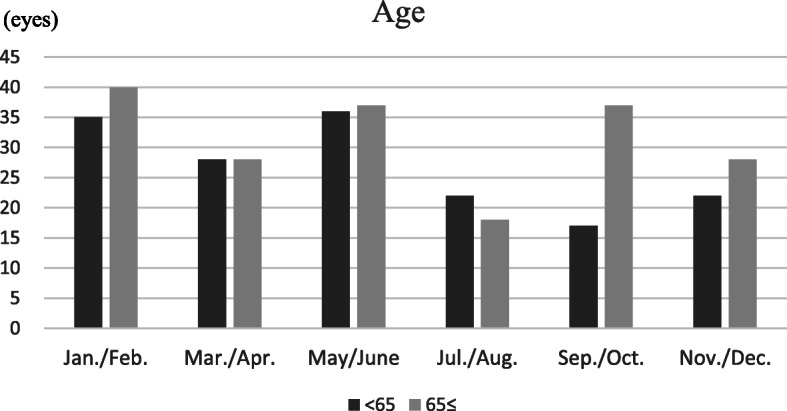
Retinal vein occlusion onset periods, with patients classified by age (dark bars for < 65 years old, grey bars for ≥65 years old)

Patients < 65 years old accounted for 160 eyes with RVO (46.0%), whereas those aged ≥65 accounted for 188 eyes (54.0%). The highest incidence of RVO among patients aged < 65 was found within the May/June period, with 36 eyes (22.5%; see Fig. 4); the lowest incidence was in the September/October period, with 17 eyes (10/6%). For patients aged ≥65, the highest incidence was found within the January/February period, with 40 eyes (21.3%; see Fig. 4); the lowest incidence was in the July/August period, with 18 eyes (9.6%). There was only marginally significant variation among six periods in patients under 65 (*p* = 0.0499), and multiple comparisons did not find the responsible seasons for the variation.

## Discussion

**Fig. 5:**
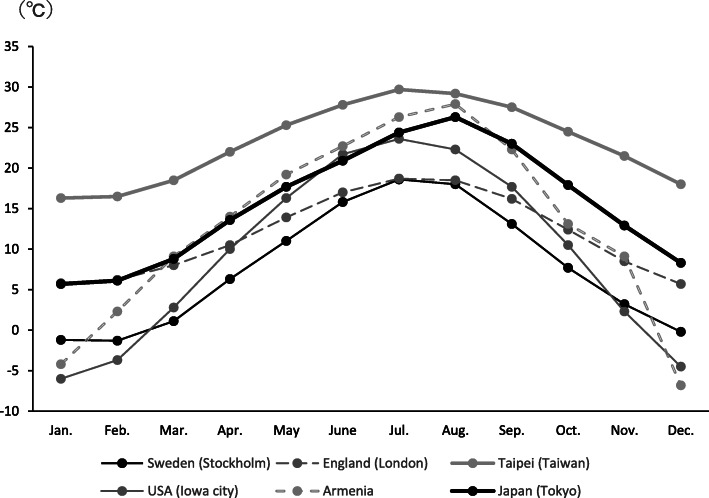
Average monthly temperatures at the study locations mentioned in the text

Here we investigated whether seasonal climatic changes affect the incidence of RVO in Japan, and found a significant variation among six 2-month periods in a year. We found increased incidence in January/February and May/June periods compared to the trough July/August period. Interestingly, statistically significant two-peak/one-trough pattern was detected only in the subpopulation of females, although similar tendency was observed for most of the subpopulations. The results in some previous studies [17–19] were similar to those in this study, with a higher incidence of RVO in winter (January/February) and a lower incidence in summer (July/August). However, studies conducted in Iowa City, Iowa, USA and (countrywide) in Armenia did not find any seasonal variation in the onset of CRVO [20, 21]. The average monthly temperatures in each of the previous study locations are shown in Fig. 5. A study investigating seasonal variation in stroke onset found that there was only an association in locations where the annual temperature differential was greater than 10 °C [16, 22] . In the aforementioned studies that found significant seasonal variation in the onset of RVO, the annual temperature differential was more than 10 °C. However, the studies conducted in Iowa City and Armenia, which did not find any seasonal variation in the onset of RVO, were also in regions that have an annual temperature differential of more than 10 °C. Therefore, the conclusion regarding the impact of the temperature differential is not universally applicable. It is anticipated that factors such as patient race and access to heating equipment could influence these findings, and future studies will need to elucidate the impact of those factors, as well as explore additional geographical regions, to clarify the influence of weather conditions on RVO.

In this study, RVO tended to develop more in January/February and May/June and less in July/August. The RVO rates may be related to low temperatures in January and February and high humidity in May and June (the rainy season in Japan). A bimodal pattern was also reported in London [18], where the first peak is observed in winter and the second peak coincides with the beginning of rainy season (September). In another vascular occlusive disease, cerebral infarction, some studies showed that its occurrence was highest in summer [9], some in winter [10]. As possible mechanisms contributing to vascular occlusion, it has been suggested that arteriosclerosis and venous obstruction are more prominent during winter, with dehydration prevalent in the summer [23]. As we found the incidence of RVO to be lowest during the summer, dehydration may not be a risk factor for the onset of this condition. In addition, the onset of cerebral hemorrhage and myocardial infarction have been found to be lower in summer and higher in winter [24, 25], which is in accordance with the findings of this study. Also, some studies show the existence of a highly significant seasonal pattern in the occurrence of DVT, characterized by a winter peak [14, 15] and DVT is associated with high vapor pressure [15]. These studies suggest that lower temperature and high vapor pressure may lead to the higher incidence of vein thrombosis. It is tempting to hypothesize that our observation of another peak in the May/June period might be due to the high vapor pressure, in spring, before reaching the high temperatures of summer. However, it needs to be determined whether this pattern of bimodal peaks is repeatedly observed, especially in other countries with rainy seasons.

Another factor that may account for the seasonal variation is blood pressure fluctuation. Hypertension has been reported to be a major risk factor for cerebral hemorrhage and myocardial infarction, and is believed to also be important for the development of RVO [4]. However, we did not found any statistically significant effect of the patients’ history of hypertension. This could be due, at least in part, to the fact that some of the patients were already on treatment for hypertension. However, this does not exclude an effect of seasonal changes in blood pressure on the incidence of RVO. For example, a negative correlation between atmospheric pressure and systolic blood pressure has been observed [26]. In addition, respiratory and circulatory system parameters, such as ventilation, heart rate, blood pressure and red blood cell count, increase when the temperature or atmospheric pressure decreases [11]. In addition to the blood pressure changes, the climate in winter has been shown to affect the parameters that explain the increased risk of arteriosclerosis, vein occlusion and hypertension during that season. For instance, catecholamines, cholesterol and vasopressin increase during winter [27]. Low temperature is associated with an increase in blood viscosity. Low temperature may also cause an increase in platelets, erythrocytes and fibrinogen, and a decrease in antithrombin III [6, 7]. It has also been suggested that lower blood levels of vitamin D in winter may be related to an increased incidence of CRVO [28].

This study had some limitations. There was a recruitment bias with our cohort, whereby patients were only eligible to be included in the study if they visited the hospital. This excluded patients who were asymptomatic or those who did not visit the hospital because of a lack of concern about their condition. Of the patients who did visit the hospital, those for whom the date of RVO onset could not be established were excluded from the study; which may also introduce some bias. In addition, the effect of cardiovascular risk factors other than hypertension, such as smoking, diabetes and hypercholesterolemia, were not investigated in our study. Finally, as this study was only conducted over a short period (48 months) and at a single facility, future studies should focus on longer-term recruitment and multiple sites.

## Conclusions

We clarified the bimodal peaks of RVO incidence in January/February and May/June periods under the climate settings of Japan. It is necessary to be vigilant about the possible occurrence of RVO in winter and the rainy season, especially for females, but regardless of the patient’s age and hypertension status. Although the mechanism remains unknown, lower temperatures and higher vapor pressure may be involved in the onset of RVO. In addition to the prevention of acute myocardial infarction and cerebral hemorrhage, people are advised to avoid acute temperature drop to prevent RVO. Future studies should examine RVO subtypes such as BRVO and CRVO, and ischemic and non-ischemic types. Such studies should also incorporate blood tests including coagulation factors, blood cell components and hormones.

## Data Availability

All data generated or analyzed during this study are available from the corresponding author on reasonable request.

## Abbreviations

RVO: Retinal vein occlusion
BRVO: Branch retinal vein occlusion
CRVO: Central retinal vein occlusion
DVT: Deep vein thrombosis
